# Green Tea Extract Induces the Resistance of *Caenorhabditis elegans* against Oxidative Stress

**DOI:** 10.3390/antiox3010129

**Published:** 2014-03-04

**Authors:** Sami Abbas, Michael Wink

**Affiliations:** 1 Department of Biotechnology, Faculty of Applied Sciences, University of Kalamoon, Damascus countryside, Deir Attiah, Syria; E-Mail: sami.abbas@uok.edu.sy; 2 Institute of Pharmacy and Molecular Biotechnology, Heidelberg University, Im Neuenheimer Feld 364, Heidelberg 96120, Germany

**Keywords:** LC/ESI-MS, catechins, antioxidants, DPPH^•^, superoxide anion radical, *hsp-16*.*2/GFP*

## Abstract

Epidemiological studies on the effects of green tea consumption (*Camellia sinensis*) have demonstrated a reduction for the risk of age-related diseases. The investigation of the *in vivo* and *in vitro* antioxidant properties of an aqueous extract of green tea (GTE) was the aim of the current study. 2,2-Diphenyl-1-picrylhydrazyl (DPPH^•^) and superoxide anion radical (O_2_^•−^) assays were used to estimate the GTE antioxidant activity. To investigate the protective effects of GTE against oxidative stress, wild-type N2 and transgenic strains (TJ374, *hsp-16*.*2/GFP*) of the model organism, *Caenorhabditis elegans* (*C*. *elegans*), were chosen. In the current study, the following catechins were identified by LC/ESI-MS: catechin, epicatechin, epicatechin gallate, gallocatechin, epigallocatechin and epigallocatechin gallate. GTE exhibited a free radical scavenging activity of DPPH^•^ and O_2_^•−^ with IC_50_ 8.37 and 91.34 µg/mL, respectively. In the *C*. *elegans* strain (TJ374, *hsp-16*.*2/GFP*), the expression of *hsp-16*.*2/GFP* was induced by a nonlethal dose of juglone, and the fluorescence density of *hsp-16*.*2/GFP* was measured. The *hsp-16*.*2/GFP* was reduced by 68.43% in the worms pretreated with 100 µg/mL GTE. N2 worms pretreated with 100 µg/mL GTE exhibited an increased survival rate of 48.31% after a lethal dose application of juglone. The results suggest that some green tea constituents are absorbed by the worms and play a substantial role to enhance oxidative stress resistance in *C*. *elegans*.

## 1. Introduction

Reactive oxygen species (ROS) are important molecules in biological systems, which are produced as by-products of normal metabolism. ROS include radical molecules, such as superoxide anion, and non-radical molecules, such as H_2_O_2_. Peroxisomes contribute to H_2_O_2_ production, which use it to oxidize a variety of molecules. On the other hand, peroxisomes contain catalase, which degrades H_2_O_2_ to H_2_O and O_2_. However, in the case of the overproduction of H_2_O_2_ or when peroxisomes are damaged, cells can become exposed to oxidative stress. Overproduction of free radicals and ROS can damage important cellular macromolecules (DNA, proteins and biomembranes), which would contribute to ROS- and age-related diseases, including cancer and neurodegenerative and cardiovascular diseases [1,2,3,4,5].

*Camellia sinensis* L. Kuntze (Theaceae) has been used for medicinal purposes for several centuries. Today, it is a widely consumed beverage throughout the world and cultivated commercially in Asia, Africa and South America [6]. Green tea and its constituents exhibit a broad array of biological activities. The most important green tea constituents are the catechins, a group of bioactive polyphenols. They include epigallocatechin gallate (EGCG), epicatechin gallate (ECG), gallocatechin (GC), epigallocatechin (EGC), epicatechin (EC) and catechin (C) [7]. EGCG is one of the major components in green tea [8], and its pharmacology has been intensively studied [9,10,11,12,13,14,15].

Green tea polyphenols showed positive effects for cardiovascular diseases and as cancer chemopreventive agents [16,17,18,19,20,21,22]. Several studies have demonstrated that green tea acts as an anti-inflammatory agent [23]. Green tea consumption can improve metabolic biomarkers [24] and lower body weight [25]. There is good evidence that the beneficial effects of green tea result from the antioxidant activities of its polyphenols, which are able to scavenge free radicals and reactive oxygen species [26,27,28]. Furthermore, polyphenols can dissociate into phenolates with O^−^ ions under physiological conditions. The hydroxyl groups and O^−^ ions can form hydrogen and ionic bonds with multiple proteins and nucleic acids in the body. This interaction influences protein flexibility, conformation and, as a consequence, their bioactivity. Therefore, they can modulate various proteins (enzymes, receptors and transcription factors) that play a role in health disorders and diseases [29,30,31].

The versatile model nematode, *C*. *elegans* Maupas (Rhabditidae), is useful for understanding the effects of pro-oxidants and antioxidants and their mode of action, because the nematodes have a rapid life cycle, short lifespan, well-established genetic pathways and are easy to cultivate [28,32]. Because *C*. *elegans* shares a substantial number of genes and pathways with humans, these nematodes have become an interesting pharmacological model organism [33,34,35].

EGCG is one of the most active and bioavailable antioxidant in green tea [36]. Our previous results showed that EGCG induces longevity and protects the model organism *C*. *elegans* against oxidative stress induced by the pro-oxidant naphthoquinone, juglone [27,28]. EGCG probably acts through the *daf2*/insulin-signaling pathway, because EGCG induces a translocation of the transcription factor DAF-16 from cytoplasm into nucleus in the TJ356 (DAF-16/GFP) strain [37]. Catechin, another constituent from green tea, can extend the lifespan in *C*. *elegans* and increase its stress resistance by modulating the energy-intensive stress response and repair system [38], which is in agreement with the Disposable Soma Theory [39]. Furthermore, the phenolics of resveratrol, aspalathin from *Aspalathus linearis* and anthocyanin-rich purple wheat increased longevity and resistance against oxidative stress in *C*. *elegans* [40,41,42]. The effects of aspalathin and anthocyanin-rich purple wheat might be mediated via a regulation of the DAF-16/FOXO insulin-like signaling pathway [40,42]. Blueberry polyphenols prolong the lifespan [43] through the CaMKII pathway, which mediates osmotic stress resistance [44]. *Ginkgo biloba* EGb761 protects the worms against oxidative damage, which accumulates upon aging [45]. Valproic acid prolongs the lifespan by inhibiting the activity of the insulin/IGF-1 signaling pathway, in addition to other targets [46].

The present study aimed to investigate the ability of an aqueous extract of green tea (GTE) to scavenge the free radicals *in vitro* and to elucidate the effect of GTE treatment on oxidative stress resistance in *C*. *elegans*. The transgenic strain, TJ374 (*hsp-16*.*2/GFP*), of the model organism, *C*. *elegans*, was used to investigate the ability of GTE to be absorbed by *C*. *elegans* and to protect the worms against oxidative stress. This strain is a useful tool to monitor the oxidative stress effects in living organism, since *hsp-16*.*2/GFP* is used as a marker for the oxidative stress. 

## 2. Experimental Section

### 2.1. Chemicals and Plant Material

Japanese green tea was purchased from Paul Schrader GmbH & Co. KG, Bremen, Germany. Sixty grams of dried leaves of green tea were immersed into 2 L of water overnight at 40 °C. The water extract was collected, filtered and finally lyophilized. For our experiments, the powder was dissolved in sterilized and bidistilled water. For *in vitro* tests, a range of GTE concentrations was used that allows one to draw linear curves and to calculate the IC_50_. For *in vivo* experiments, the GTE concentration is chosen depending on the curves resulting from *in vitro* study, since the concentration of 100 µg/mL of GTE was almost in the plateau part of the curves (Figure 3). (−)-Epigallocatechin gallate (EGCG) 95%, 2,2-diphenyl-1-picrylhydrazyl (DPPH^•^), nicotinamide adenine dinucleotide (NADH), phenazine methosulfate (PMS) and 5-hydroxy-1, 4-naphthoquinone (juglone) were obtained from Sigma-Aldrich GmbH (Steinheim, Germany). Nitroblue tetrazolium (NBT) and sodium azide came from AppliChem GmbH (Darmstadt, Germany).

### 2.2. *C. elegans* Strains and Culture Conditions

The TJ375 (*hsp-16*.*2/GFP*) and N2 strains were obtained from the *Caenorhabditis* Genetic Center (CGC), which was founded by NIH National Center for Research Resources. The strains were maintained at 20 °C on solid nematode growth medium (NGM) [32]. All worms used in this study were age-synchronized, and the experimental animals were grown in liquid S-medium and raised from eggs obtained by sodium hypochlorite treatment of hermaphrodites [47].

### 2.3. Analysis of Catechins in GTE Using the LC/ESI-MS

The HPLC system consists of a degasser, Merck-Hitachi L-6200A low pressure binary pumps and Lichro-CART RP-C18_e_ column (250 × 4.6 mm i.d.; 5 µm) with a compatible guard column. The mobile phase was operated under gradient condition with 0.5% aqueous acetic acid (A) and acetonitrile (B), at a flow rate 1 mL/min at room temperature as follows: 0–60 min 0%–40% B, 60–70 min 40%–100% B. The MS machine used was a VG Quattro II model with an electrospray source and a quadrupole analyzer. Acquisition was done in negative mode using single-quad with the following parameters: drying and nebulizing gas (N_2_) with a flow rate of 3.5 L/h and 350 L/h, respectively; source temperature, 120 °C; scan range, 200–1000 *m*/*z*; cone voltage, 30 V; focusing voltage, 0.5 V; and capillary voltage, 3.5 kV.

### 2.4. DPPH^•^ Free Radical Scavenging Activity

The free radical scavenging activity of GTE was measured using 2,2-diphenyl-1-picrylhydrazyl (DPPH^•^), following the standard methods [48,49,50] with some modifications. The antioxidants reduce the stable free radical DPPH^•^ (deep violet) to the diphenyl-picrylhydrazine DPPH-H (pale yellow). The DPPH^•^ molecule is a stable free radical characterized by an absorption band at about 517 nm. As the odd electron of the radical becomes paired in the presence of a hydrogen donor (antioxidant), the absorption strength is decreased [49,51]. Zero-point-two millimolar of DPPH^•^ in methanol was prepared, and 500 µL of this solution was added to 500 µL of GTE at different concentrations that allow one to draw linear curves and to calculate the IC_50_. Thirty minutes later, the absorbance was measured at 517 nm. The scavenging activity of the DPPH^•^ free radicals (in %) was calculated using the following equation:

DPPH^•^ scavenging effect (%) = [(*A*_0_ − *A*_1_)/*A*_0_] × 100 (1)

*A*_0_ is the absorbance of the control reaction (DPPH^•^ reagent without sample), and *A*_1_ is the absorbance in the presence of GTE. EGCG was employed as a positive control. Data were obtained from three independent experiments. The mean and standard error were calculated for each concentration.

### 2.5. Superoxide Anion Radical Scavenging Activity

The superoxide anion scavenging activity of GTE was determined by the method of Robak and Gryglewski [52]. Superoxide anion radicals are generated in the PMS-NADH system by oxidation of NADH and assayed by the reduction of NBT. The superoxide radicals were generated in 900 µL of 0.1 M phosphate buffer, pH 7.4 (reference), or 800 µL (test), 100 µL water (control) or GTE at different concentrations that allow one to draw linear curves and to calculate the IC_50_, 100 µL NADH (156 µM) and 100 µL NBT (630 µM). The reaction starts by adding 100 µL PMS (30 µM) to the mixture. After 5 min of incubation at 25 °C, the absorption was measured at 560 nm. The percentage of inhibition of superoxide anion generation was calculated using the following equation:

O_2_^−•^ scavenging effect (%) = [(*A*_0_ − *A*_1_)/*A*_0_] × 100 (2)

*A*_0_ is the absorbance of the control reaction, and *A*_1_ is the absorbance in the presence of GTE. EGCG was employed as a positive control. Data were obtained from three independent experiments. The mean and standard error were calculated for each concentration.

### 2.6. Quantitation of *hsp-16.2/GFP* Expression in *C. elegans*

TJ375 (*hsp-16*.*2/GFP*) worms were cultivated in liquid S-medium at 20 °C, and *E*. *coli* (OP50) was added to the media (1 × 10^9^ cell/mL) as a food source. *hsp-16*.*2* is expressed by either heat shock or by pro-oxidative stress, like exposure to the naphthoquinone, juglone [53]. The expression of *hsp-16*.*2* was measured directly by observing the fluorescence of the green fluorescent protein (GFP) as the reporter protein. The worms were treated with 100 µg/mL of GTE on the day after hatching for 48 h. Worms were transferred to new medium, and oxidative stress was then exerted by adding 20 µM juglone to the medium for 24 h. After induction, worms from each set of experiments were mounted onto a glass slide in M9 medium containing 10 mM sodium azide to reduce their mobility [47]. Images of fluorescence were taken at constant exposure times using the 20× objective (Nikon-eclipse 90i, Nikon Imaging Center, Heidelberg University) with a digital camera (Nikon digital sight DS-Qi1Mc). The images included the anterior part of worms from the back of the pharynx. For quantifying the GFP fluorescence, images were analyzed using ImageJ software. The image of the anterior part of the worms was outlined, black to white inverted and the mean pixel density measured.

### 2.7. Survival Assay

Through induced oxidative stress, a higher concentration of juglone (80 µM) is lethal. N2 worms were maintained at 20 °C and treated with 100 µg/mL GTE on the day after hatching for 48 h, and then, worms were transferred to new medium. Eighty micromolar juglone was added to the medium, and the survivors were counted after 24 h of juglone treatment.

### 2.8. Statistical Analyses

Statistical comparison between controls and treatments were done with two-tailed unpaired student’s *t*-test, assuming equal variance. All figures indicate means and the standard error of the mean. Data were obtained from three independent experiments. Error bars represent the standard error (SE). *p* < 0.05 was considered for significant differences. 

## 3. Results and Discussion

### 3.1. Catechins of GTE

**Figure 1:**
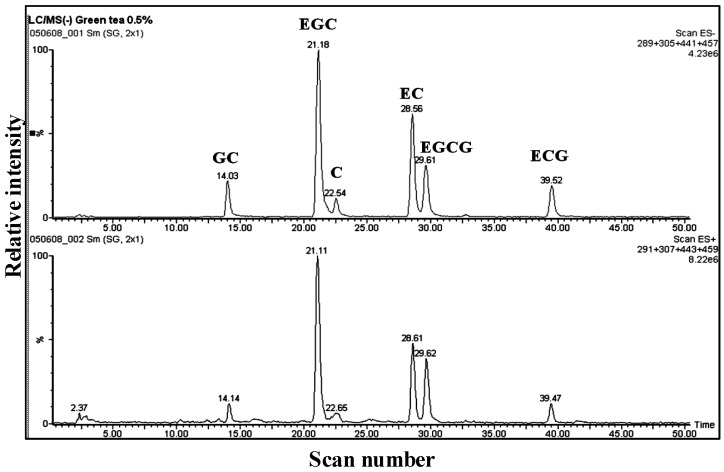
LC/ESI-MS analysis of aqueous extract of green tea (GTE). Reconstructed ion chromatogram (RIC) obtained in both positive and negative scan modes. GC, gallocatechin; EGC, epigallocatechin; C, catechin; EC, epicatechin; EGCG, epigallocatechin gallate; ECG, epicatechin gallate.

**Figure 2:**
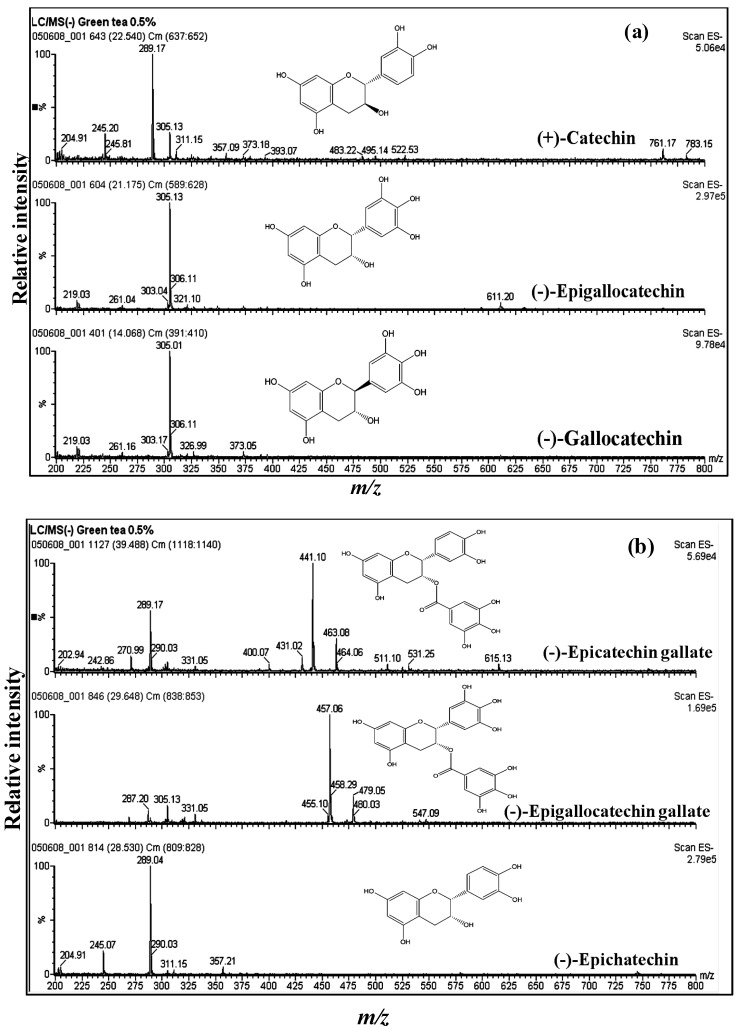
Mass spectra of catechins. (**a**) Catechin with [M − H]^–^ of *m/z* 289, epigallocatechin with [M − H]^–^ of *m/z* 305 and gallocatechin with [M − H]^–^ of *m/z* 305; (**b**) epicatechin gallate with [M − H]^–^ of *m/z* 441, epigallocatechin gallate with [M − H]^–^ of *m/z* 457 and epicatechin with [M − H]^–^ of *m/z* 289.

Several studies have applied LC/ESI-MS to identify the main secondary metabolites of green tea, especially of catechins (flavan-3-ols), using retention time and mass spectral information [7,54,55,56]. A reconstructed ion chromatogram (RIC) of the aqueous extract is shown in Figure 1. MS analysis allowed the identification of catechins by the presence of informative [M − H]^−^ ions, along with a unique retention time for each component. The mass spectrum of EGCG shows a fragment ion at *m/z* 287 resulting from the loss of water from the EGC moiety, and *m/z* 305 represents the EGC moiety (Figure 2b, Table 1). The mass spectrum of ECG shows a fragment ion at *m/z* 271 resulting from the loss of water from the epicatechin moiety (Figure 2b, Table 1). Mass spectral data are partly in agreement with the previous results of Poon [55], because he obtained the full scan negative ionization mass spectra, while in our case, the scan range was 200–1000 *m/z*. Figure 2a,b illustrates the mass spectra and structures of catechin, epicatechin, gallocatechin, epigallocatechin, epicatechin gallate and epigallocatechin gallate with [M − H]^−^ at *m/z* 289, 289, 305, 305, 441 and 457, respectively; these findings are in agreement with published results [7,54]. The molecular ions and some informative fragments of GTE catechins are summarized in Table 1. 

**Table 1:** Molecular ions and some significant fragments of main catechins presented in GTE.

Compound	MW	[M − H]^−^	Significant fragments
Catechin (C)	290	289	
Epicatechin (EC)	290	289	
Gallocatechin (GC)	306	305	
Epigallocatechin (EGC)	306	305	
Epicatechin gallate (ECG)	442	441	*m/z* 271 (EC without H_2_O) *m/z* 289 (EC)
Epigallocatechin gallate (EGCG)	458	457	*m/z* 287 (EGC without H_2_O) *m/z* 305 (EGC)

### 3.2. GTE Scavenges DPPH^•^ and O_2_^•−^ Free Radicals *in Vitro*

**Figure 3:**
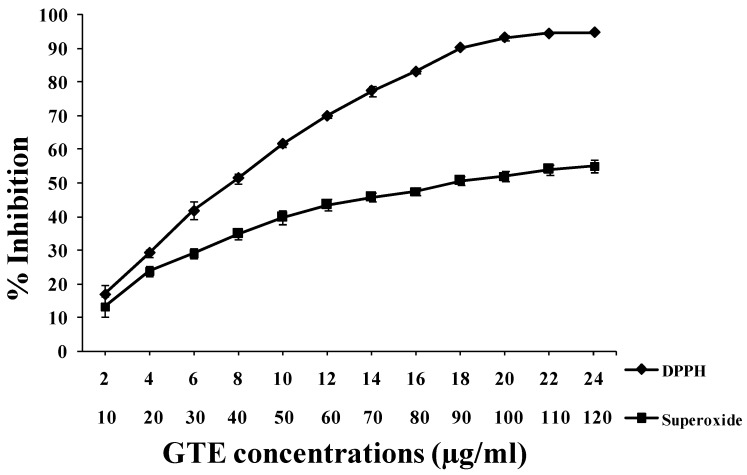
Two assays were used to measure the *in vitro* antiradical activities of GTE. Free radical scavenging activity was determined by the 2,2-diphenyl-1-picrylhydrazyl (DPPH^•^) assay and superoxide anion radical assay. The superoxide anion radicals were generated in the phenazine methosulfate (PMS)-nicotinamide adenine dinucleotide (NADH) system by oxidation of NADH and assayed by the reduction of nitroblue tetrazolium (NBT). The results of these tests are expressed as the percent inhibition of the control (reagent without sample) that is calculated by Equations (1) and (2). For the DPPH^•^ assay, the range of GTE concentrations used was 2–24 µg/mL. For the superoxide assay, the range of GTE concentrations used was 10–120 µg/mL. Data were obtained from three independent experiments. The mean and standard error were calculated for each concentration. IC_50_ values were 8.37 ± 0.14 and 91.34 ± 1.98 µg/mL for DPPH^•^ and superoxide anion radicals, respectively.

The excessive oxidation reactions may occur from exposure to stress, smoking and chemical reagents, resulting in the production of free radicals that can contribute to the onset of various disease states, such as cardiovascular diseases, cancer and neurological disorders, and also accelerate aging [1,2,57,58,59]. GTE showed a strong free radical scavenging activity with an IC_50_ of 8.37 ± 0.14 µg/mL (Figure 3). The superoxide anion was generated in a non-enzymatic system using PMS, NADH and NBT. The decrease of absorbance at 560 nm with GTE indicates a consumption of superoxide anion in the reaction mixture with an IC_50_ of 91.34 ± 1.98 µg/mL (Figure 3). These results are in agreement with our earlier studies on EGCG [27] and those of other reports [8,31,60,61,62,63].

### 3.3. GTE Reduces the Stress-Induced *hsp-16-2* Expression in *C. elegans*

**Figure 4:**
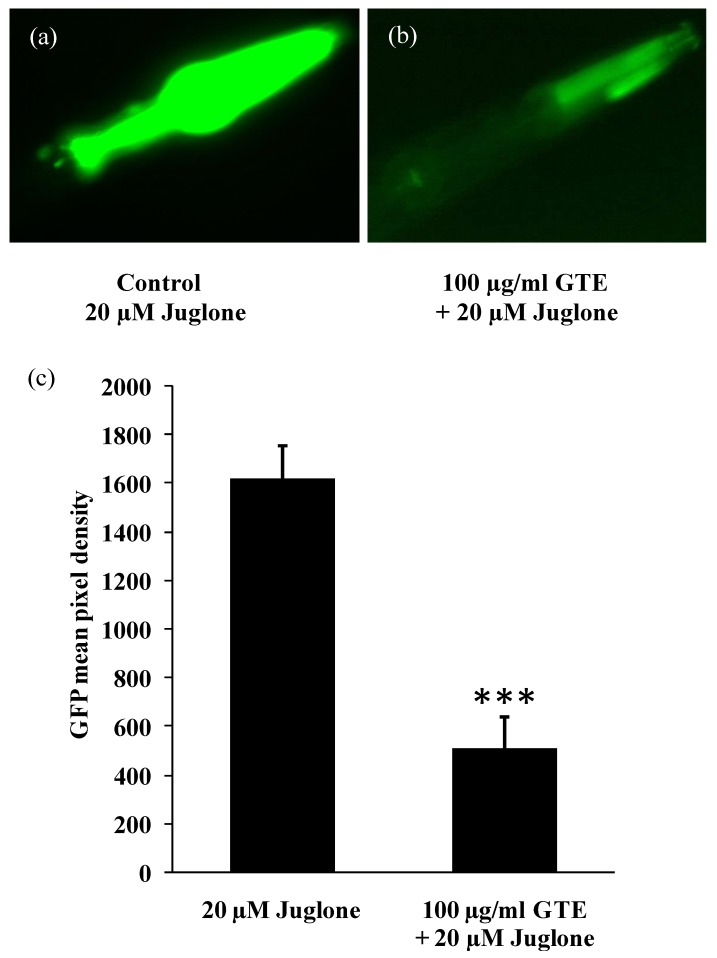
*C*. *elegans* strain TJ375 (*hsp-16*.*2/GFP*) visualizes the induction of the *hsp-16*.*2/GFP* reporter in response to juglone treatment. Experimental worms were cultured in liquid medium and treated without (**a**) and with (**b**) 100 µg/mL GTE for 48 h followed by 20 µM juglone (juglone treatment was in new medium) for 24 h. (**c**) The quantitation of the GFP in response to oxidative stress. The GFP fluorescence is not detectable with GTE alone or with no juglone treatment. Comparisons between treatments and controls were significant using the two-tailed, unpaired Student’s *t*-test. Data were obtained from three independent experiments with 25 worms in each group. *** *p* *<* 0.001.

Small heat shock proteins (sHSPs) are biomarkers for the exposure to thermal and oxidative stressors, and they are highly inducible during exposure to pro-oxidants, such as juglone [53]. sHSPs act as molecular chaperones by preventing the accumulation of aggregated proteins [64]. The TJ375 strain has a GFP reporter controlled by the *hsp-16*.*2* promoter [53] that is not active under standard conditions. However, after exposure to oxidative stress, *hsp-16*.*2* becomes induced, and the worms exhibit an extensive GFP expression in the pharynx (Figure 4). In the present study, the TJ375 strain is used to figure out the protective effect of GTE against oxidative stress produced by treatment with nonlethal dose of juglone. The expression of *hsp-16*.*2/GFP* (induced by 20 µM juglone) was suppressed by 68.43% in worms fed with 100 µg/mL of GTE, as compared to controls (control: GFP mean pixel density of 1,621.644 ± 133.22 *vs*. GTE, GFP mean pixel density of 512.01 ± 126.89, *p* < 0.001; Figure 4). These results indicate that GTE is bioavailable and able to increase the stress resistance in *C*. *elegans*. 

### 3.4. GTE Increases Survival Rate in *C. elegans*

**Figure 5:**
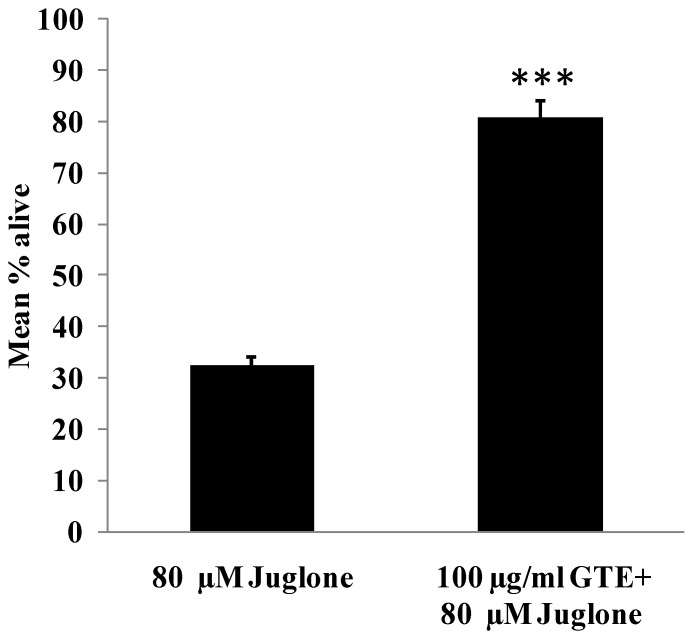
Survival rates of wild-type *C*. *elegans* under an acute lethal dose of juglone. In this experiment, the worms were cultured in liquid medium and treated with 100 µg/mL GTE for 48 h followed with 80 µM juglone for 24 h (juglone treatment was in new medium). The mean percentage of live worms was calculated from three independent experiments. Comparisons between treatments and controls were significant using the two-tailed, unpaired, Student’s *t*-test. Data were obtained from three independent trials, each consisting of at least 150 worms. *** *p* *<* 0.001.

In this experiment, N2 worms were pretreated with 100 µg/mL GTE for 48 h and then transferred to new medium and exposed to a lethal dose of juglone (80 µM) for 24 h, after which the alive worms were counted. The results (Figure 5) showed that the pretreatment of the N2 worms with 100 µg/mL GTE is indeed able to protect the organism against lethal oxidative stress. The average of surviving worms after treatment with 80 µM juglone was 32.43 ± 1.71% and for the group pretreated with GTE for 48 h was 80.74 ± 3.51% (the increase rate was 48.31%, *p* < 0.001, Figure 5). 

The treatment of worms with EGCG showed similar results by upregulating the aging-associated genes, such as *daf-16*, *sod-3*, and *skn-1* [65]. Compared with EGCG, the mode of action of GTE seems to be similar, since EGCG activates the *daf2*/insulin-signaling pathway by the induction of DAF-16 translocation from cytoplasm into nucleus in the TJ356 (DAF-16/GFP) strain [37]. The inhibition of *daf-2*, a homologue of the insulin receptor, leads also to nuclear localization of DAF-16/GFP [66]. DAF-16/FOXO is a transcription factor that promotes longevity and stress resistance, including antioxidative enzymes, like superoxide dismutase and catalase [67]. In addition, several studies showed that polyphenols can enhance oxidative stress resistance and prolong the lifespan in *C*. *elegans*, probably by modulation of the *daf2*/insulin-signaling pathway [37,40].

## 4. Conclusions

Catechins represent 30% of the dry weight of green tea leaves and are the active components of GTE, which exhibits considerable antioxidant activities *in vitro* and *in vivo*. In *C*. *elegans*, GTE reduces the oxidative stress and elevates the stress resistance by attenuating the heat shock protein (*hsp-16*.*2*) response and by increasing the survival rate after exposition to a lethal dose of the pro-oxidant, juglone. Our results imply that the epidemiological data, which see the beneficial effects of green tea consumption with good health and the reduction of the risk of several diseases related to oxidative stress, have a rational base.
